# Two asymptomatic facial erythematous macules in an adult male

**DOI:** 10.1002/ski2.172

**Published:** 2022-10-06

**Authors:** Kamran Balighi, Kambiz Kamyab‐hesari, Parvaneh Hatami, Zeinab Aryanian

**Affiliations:** ^1^ Autoimmune Bullous Diseases Research Center Tehran University of Medical Sciences Tehran Iran; ^2^ Department of Dermatology School of Medicine Razi Hospital Tehran University of Medical Sciences Tehran Iran; ^3^ Department of Dermatopathology School of Medicine Razi Hospital Tehran University of Medical Sciences Tehran Iran; ^4^ Department of Dermatology Babol University of Medical Sciences Babol Iran

## Abstract

Pemphigus is a group of autoimmune bullous disorders with different types. Pemphigus foliaceous (PF) is a difficult‐to‐diagnosis disorder which shares clinical features with many dermatoses. We hereby, present an interesting case of PF which serves as a reminder for clinicians that pemphigus is not always a serious condition with rapid extension of lesions and it could have a very limited and benign form.

## Graphical abstract

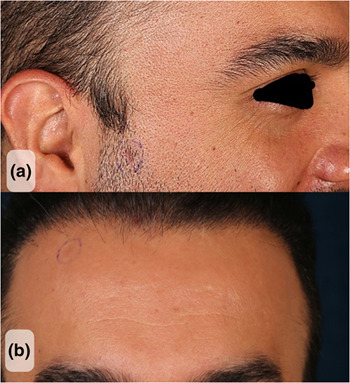

## 1 CASE PRESENTATION

**FIGURE 1:**
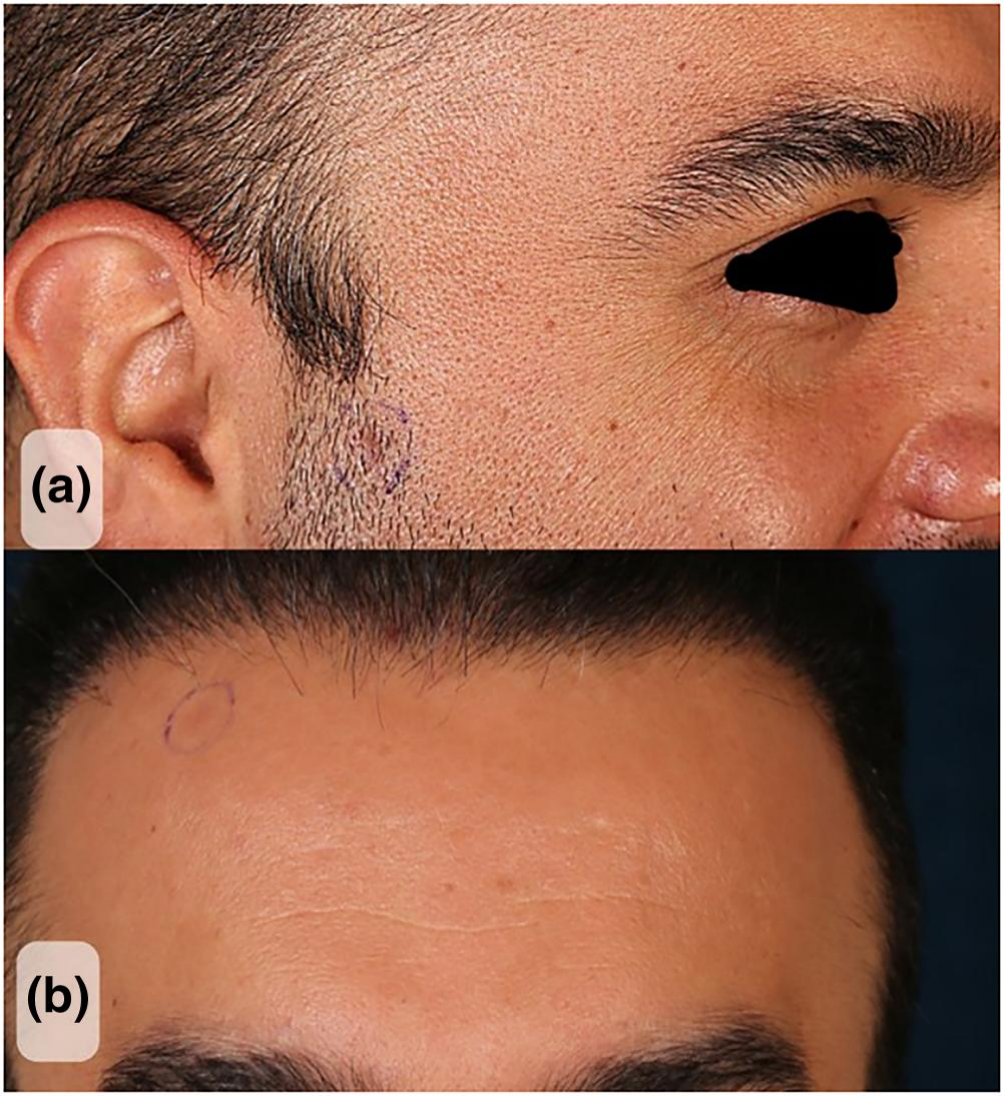
Two small erythematous macules on the right cheek (a) and forehead (b) area

A 32‐year‐old male presented to the dermatologic clinic with two asymptomatic facial lesions without any change in size over the course of the past 12 months (Figure 1). The patient did not have any other skin eruptions, mucosal involvement or photosensitivity and any remarkable past medical history as well as occupational or family history.

Physical examination revealed two erythematous macules with approximate diameter of 5 mm on the forehead and beard area. We considered all of the differential diagnoses for facial annular erythematous lesions including discoid lupus erythematosus (DLE), sarcoidosis, granuloma annularis and seborrhoeic dermatitis. The histopathologic examination and direct immunofluorescence (DIF) assay were performed.

Skin biopsy from cheek lesion revealed Prominent changes within the superficial epidermis and acanthloysis was seen mainly within the stratum granulosum.

**FIGURE 2:**
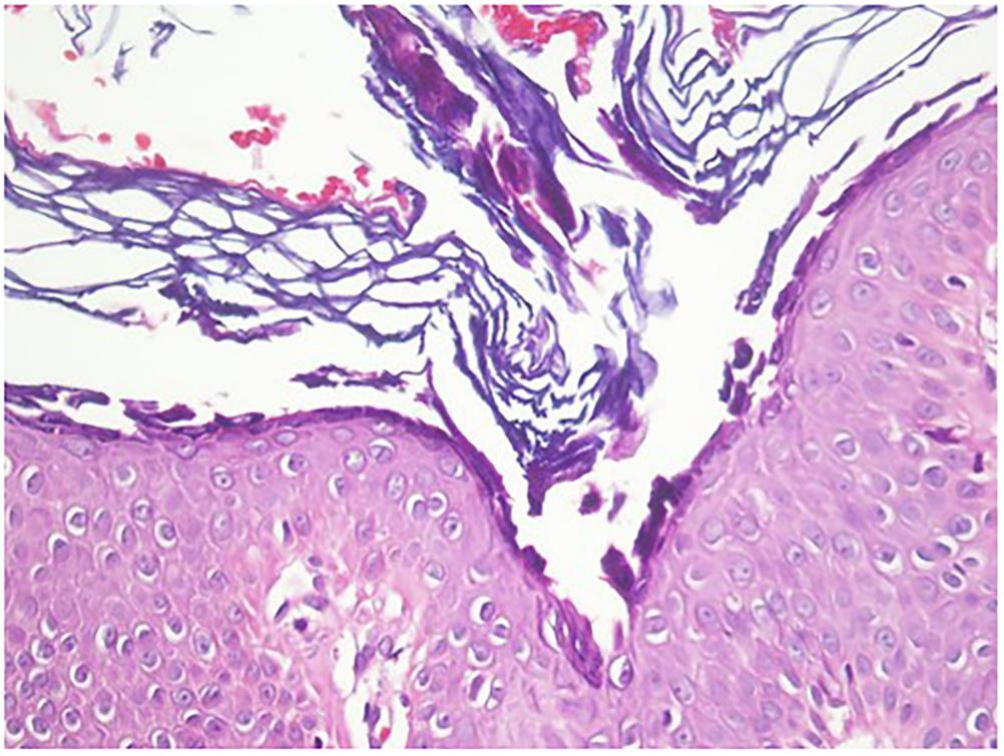
Subcorneal cleavage and acantholysis

In the dermis, there was a predominantly superficial lymphocytic infiltrate with scattered scarce eosinophils (Figure 2). DIF study showed an intercellular deposit of IgG and C3 at the epidermis.

## 2 DISCUSSION

Pemphigus is a group of autoimmune bullous disorders with different types including pemphigus vulgaris (PV), Pemphigus foliaceous (PF), Paraneoplastic pemphigus, IgA pemphigus and Drug‐induced pemphigus.^1^, ^2^ Pemphigus foliaceous is the second most common pemphigus behind pemphigus vulgaris, which occurs as a result of anti‐desmoglein‐1 antibodies production against the intercellular adhesion DSG1 protein leading to acantholysis in the stratum granulosum and characterized by skin involvement, primarily at seborrhoeic areas, without any mucosal lesions.^3^ Pemphigus foliaceus is classically presented with fragile loose blisters over seborrhoeic areas such as face, scalp and upper trunk.^4^ There are several triggering factors for the evolution of pemphigus which include medications, vaccines, genetic predisposition, pregnancy and stress.^4^

As mentioned before, PF is classically presented with fragile loose blisters over seborrhoeic areas such as face, scalp and upper trunk. The differentiating clinical characteristic of PF from pemphigus vulgaris (PV) is the mucous membrane sparing and extensive erythema in the former.^4^ However, demonstrating separation of keratinocytes at the granular layer of the epidermis, pre‐acantholytic vacuole formation in intercellular spaces and subcorneal blisters in the histopathologic examination confirms the diagnosis of PF.^5^ Direct immunofluorescence (DIF), demonstrating intercellular deposition of IgG and C3, and indirect immunofluorescence microscopy for detecting serum autoantibodies against DSGs are highly sensitive and specific methods for diagnosing pemphigus, as aided us significantly for the patient.^6^

This case presented with two small, hard to notice, asymptomatic macules. Hence, diagnosing PF could be challenging due to its non‐specific clinical presentation which might lead to a delay in correct diagnosis. Differential diagnosis of facial macules and plaques is usually made with lichen planus, DLE, pseudolymphoma, sarcoidosis and annular granuloma which were either considered for our case. However, histopathologic assessment surprisingly revealed some characteristic features of PF.

Systemic corticosteroids are still the mainstay and first‐line treatment of pemphigus. However, in moderate‐to‐severe cases, oral azathioprine or a mycophenolate compound is added.^7^ Based on the benign and limited course of disease in our patient, a topical corticosteroid was initiated for him, which led to complete resolution of skin lesions in 2 months.

This interesting case report high bolds the fact that PF might mimic various more common dermatoses, in particular when it appears as a few small lesions with a benign manner.

The serologic assay in this case regarding both anti Dsg‐1 and anti Dsg‐3 was negative which explains the benign course of disorder without developing any new lesion over last 12 months.

Because of facial involvement in this case, Senear‐Usher syndrome also known as pemphigus erythematosus, was also considered which is an overlap syndrome with features of lupus erythematosus (LE) and pemphigus foliaceus. However, histological and DIF evaluations were not consistent with this diagnosis.

This case report showed that pemphigus is not always a serious condition with rapid extension of lesions and it could have a very limited and benign form. However, owing to unpredictable course of autoimmune disorders and few case reports of patients with an established diagnosis of PF developing pemphigus vulgaris later in the course of their disease,^8^, ^9^, ^10^ long‐term follow up of this patient and those with similar features is strongly recommended.

In conclusion, PF is a difficult‐to‐diagnosis disorder which shares clinical features with many dermatoses. Clinical suspicion is necessary to ensure correct diagnosis and start the appropriate treatment.

## CONFLICTS OF INTEREST

The authors declare that they have no conflict of interest regarding the publication of this article.

## AUTHOR CONTRIBUTIONS

**Kamran Balighi**: Conceptualization; Equal, Supervision; Equal, **Kambiz Kamyab‐hesari**: Investigation; Equal, Methodology; Equal, **Zeynab Aryaniyan**: Data curation; Equal, Writing – original draft; Equal, **Parvaneh Hatami**: Investigation; Equal, Writing – review & editing; Equal.

## ETHICS STATEMENT

The patients provided written informed contest to publication of this case report and accompanying images.

## Data Availability

The data that support the findings of this study are available from the corresponding author, upon reasonable request.
